# Case report: Concurrent primary thyroid MALT lymphoma and lymph node metastatic thyroid micropapillary carcinoma in Hashimoto’s thyroiditis: a diagnostic and therapeutic challenge

**DOI:** 10.3389/fonc.2026.1848147

**Published:** 2026-07-13

**Authors:** Yuting Zhu, Qi Shi, Haoyi Su, Ben Zhang, Tongyue Zhang, Shaoxiong Lu, Tieshi Luowu, Huaiyuan Sun, Limei Qu, Qiang Zhang

**Affiliations:** 1 Department of Thyroid Surgery, General Surgery Center, The First Hospital of Jilin University, Changchun, China; 2 Department of Pathology, The First Hospital of Jilin University, Changchun, China

**Keywords:** case report, coexistence, Hashimoto’s thyroiditis, lymph node metastasis, MALT lymphoma, papillary thyroid microcarcinoma, primary thyroid lymphoma

## Abstract

**Background:**

Primary thyroid lymphoma (PTL) is an extremely rare malignancy, and its prognosis primarily depends on factors such as histological type and clinical stage. Papillary thyroid carcinoma(PTC) is the most common pathological type of thyroid cancer. Papillary thyroid microcarcinoma(PTMC) accounts for a significant proportion of PTCs and generally has a favorable prognosis;however, central lymph node metastasis (CLNM) occurs in approximately 10-30% of cases. Thecoexistence of PTL and metastatic PTMC is exceedingly rare, with only a few reports in the literature. Hashimoto thyroiditis (HT) is a common risk factor for both PTL and PTC.

**Methods:**

We present a case of HT diagnosed concurrently with PTL and PTMC, accompanied by level VI CLNM. We systematically reviewed the patient’s clinical presentation, imaging findings, laboratory results, histopathological features, treatment, and follow-up.

**Results:**

A 40-year-old woman presented with neck discomfort. Ultrasonography revealed a solid hypoechoic nodule in the right thyroid lobe with irregular and slightly lobulated margins. The patientdeclined fine-needle aspiration cytology and opted for direct surgery. The patient underwent right thyroid lobectomy with isthmusectomy and biopsy of the right central compartment (level VI) lymph nodes. Postoperative pathology confirmed the coexistence of extranodal marginal zone lymphoma of mucosa-associated lymphoid tissue (MALT lymphoma), with pathological features suggestive of large B-cell transformation, and PTMC, with carcinoma identified in one of ten resected level VI lymph nodes (1/10). Immunohistochemistry showed that the lymphoma cells were positive for CD20, CD79a, Pax-5, and Bcl-2 and negative for CD5, CD10, and Cyclin D1. The patient received four cycles (six infusions) of a single-agent, obinutuzumab, as post-surgical therapy. The patient has now completed the full treatment course, remains in a stable condition, and shows no evidence of recurrence.

**Conclusion:**

The coexistence of PTL and PTMC is exceptionally rare and poses a significant risk of misdiagnosis. Comprehensive preoperative evaluation and precise histopathological examination are crucial for accurate diagnosis. Although PTMC generally has an excellent prognosis, central lymph node metastasis can occur in a subset of patients. Careful evaluation of suspicious cervical lymph nodes and individualized postoperative surveillance are therefore warranted. Therapeutic decision-making requires a balanced consideration of lymphoma stage and thyroid carcinoma risk stratification, underscoring the importance of a multidisciplinary approach.

## 1 Introduction

Thyroid cancer is the most prevalent malignant endocrine tumor (1), with papillary thyroid carcinoma (PTC) accounting for 84% of all thyroid malignancies (2). Papillary thyroid microcarcinoma (PTMC) is defined as PTC with a maximum diameter of ≤1 cm, and the widespread use of high-resolution ultrasonography has led to a significant increase in its detection rate (3). PTMC is associated with a favorable prognosis, with a 5-year relative survival rate of 98.5% after adequate treatment (2). In contrast, primary thyroid lymphoma (PTL) is an exceedingly rare malignant tumor of the thyroid that accounts for approximately 2% of all extranodal lymphomas (4). The most prevalent histological type is diffuse large B-cell lymphoma (DLBCL), comprising approximately 50-70% of cases, while extranodal marginal zone lymphoma of mucosa-associated lymphoid tissue (MALT lymphoma) accounts for 10-50% (5). PTL typically presents as a rapidly enlarging neck mass accompanied by cervical lymphadenopathy and predominantly affects middle-aged and elderly women. It may also manifest with compressive symptoms, such as dyspnea, dysphagia, and hoarseness (6). Patients with this condition may also present with B-like symptoms. PTL is a highly heterogeneous disease whose prognosis is significantly influenced by factors including histological subtype, clinical stage, and treatment modalities. For instance, PTL of the MALT lymphoma subtype exhibits a more indolent behavior, and patients with this subtype generally experience a more favorable clinical course and superior prognosis compared to those with the DLBCL subtype (7). Early diagnosis, precise pathological classification, and comprehensive treatment with chemotherapy/immunochemotherapy are pivotal for achieving a favorable prognosis. Patients with Hashimoto’s thyroiditis (HT) have a 40- to 80-fold higher risk of developing PTL compared to the general population (8). Thyroid MALT lymphoma is widely recognized as a serious complication of HT. HT is also a widely recognized risk factor for PTC (9). In clinical practice, a substantial proportion of patients with PTMC have concurrent HT. However, some studies suggest that HT may act as a protective factor against lymph node metastasis in PTC, with these patients experiencing more favorable outcomes than those without HT (10). The co-occurrence of primary thyroid lymphoma and PTMC with central lymph node metastasis is extremely rare. Previously reported cases of concurrent primary thyroid lymphoma and PTC/PTMC are summarized in **Supplementary Table 1** (11–18). This report presents a histologically confirmed case of concurrent primary thyroid MALT lymphoma and PTMC with CLNM. This unusual combination highlights the diagnostic and therapeutic complexity of managing concurrent thyroid malignancies in the setting of HT, particularly when PTMC is accompanied by central lymph node metastasis.

## 2 Case description

**Figure 1:**
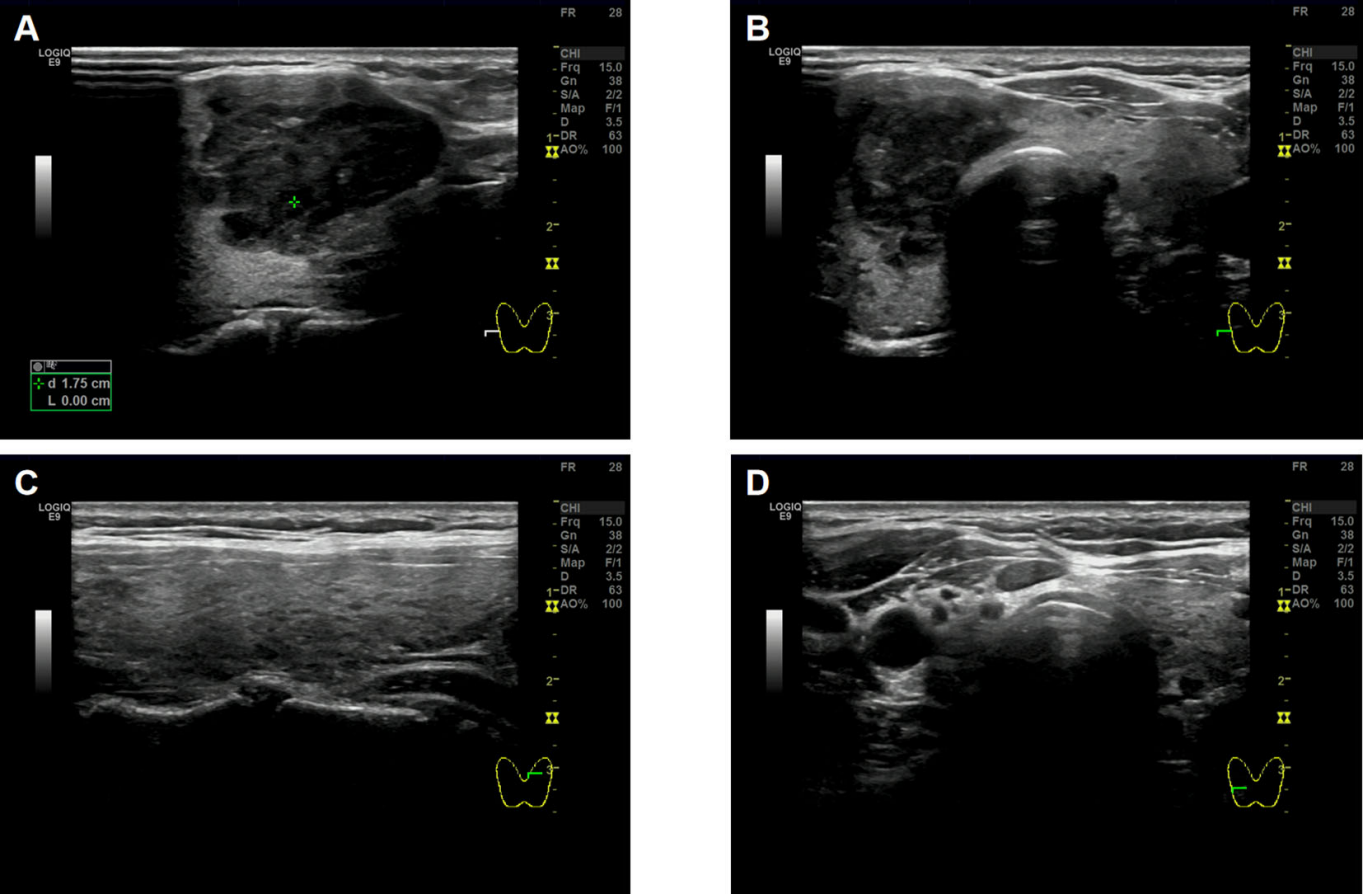
Preoperative thyroid and cervical ultrasound findings. **(A)** A nodule in the right thyroid lobe with ill-defined margins and irregular shape, measuring approximately 29 mm in diameter. **(B)** The thyroid gland exhibits heterogeneous hypoechogenicity with coarsened echotexture. **(C)** A small nodule in the left thyroid lobe with well-defined margins and regular shape, measuring approximately 3.5 mm in diameter, categorized as C-TIRADS 3. **(D)** A hypoechoic lymph node was noted at level VI of the right neck.

A 40-year-old woman presented at a local hospital with neck discomfort. An enlarged thyroid gland was observed; however, no specific treatment was initiated. The patient was referred to the thyroid surgery outpatient clinic of our hospital for further evaluation. Specialist thyroid examination revealed a palpable, firm, and non-tender mass in the right thyroid lobe, approximately 3 cm in size, that moved with swallowing. No thyroid bruits were audible on auscultation, and no significantly enlarged cervical lymph nodes were palpable. Cervical ultrasonography revealed diffuse parenchymal changes in the thyroid gland. A nodule measuring approximately 29 × 20 mm was identified in the mid-lower portion of the right lobe (C-TIRADS 4B). It appeared as a solid, hypoechoic nodule with irregular and slightly lobulated margins (**Figures 1A, B**). Another nodule was located in the midportion of the left thyroid lobe, measuring approximately 3.5×2.8 mm (C-TIRADS 3). The tumor had well-defined margins and a regular shape (**Figure 1C**). A hypoechoic lymph node, measuring about 7.4×3.4 mm with an indistinct hilum, was observed in the right level VI region, its nature undetermined (**Figure 1D**). Preoperatively, the patient had normal thyroid function, with thyroid-stimulating hormone, free triiodothyronine, and free thyroxine levels within normal limits. However, elevated levels of anti-thyroglobulin antibody (38.80 IU/mL; reference range, 0-4. 11) and anti-thyroid peroxidase antibody (310. 19 IU/mL; reference range, 0-5.61) were detected. Routine blood tests and chest radiography revealed no other abnormalities. The patient had no history of smoking, alcohol consumption, or radiation exposure. There was no family history of tumors or thyroid disease.

Based on these findings, the preliminary diagnosis was a mass in the right thyroid lobe of an undetermined nature, with particular suspicion for thyroid carcinoma with CLNM and HT. Ultrasound-guided fine-needle aspiration biopsy was recommended for definitive diagnosis, but the patient declined and opted for direct surgical intervention. The extent of surgery was determined on the basis of the available preoperative findings and the patient’s preference. Although the dominant right-lobe lesion was sonographically suspicious, no cytological or histological diagnosis was available because the patient declined preoperative FNA or core needle biopsy. The contralateral thyroid nodule was very small, measuring 3.5×2.8 mm, and was classified as C-TIRADS 3. The right level VI lymph node was indeterminate on ultrasonography rather than biopsy-proven metastatic disease.

**Figure 2:**
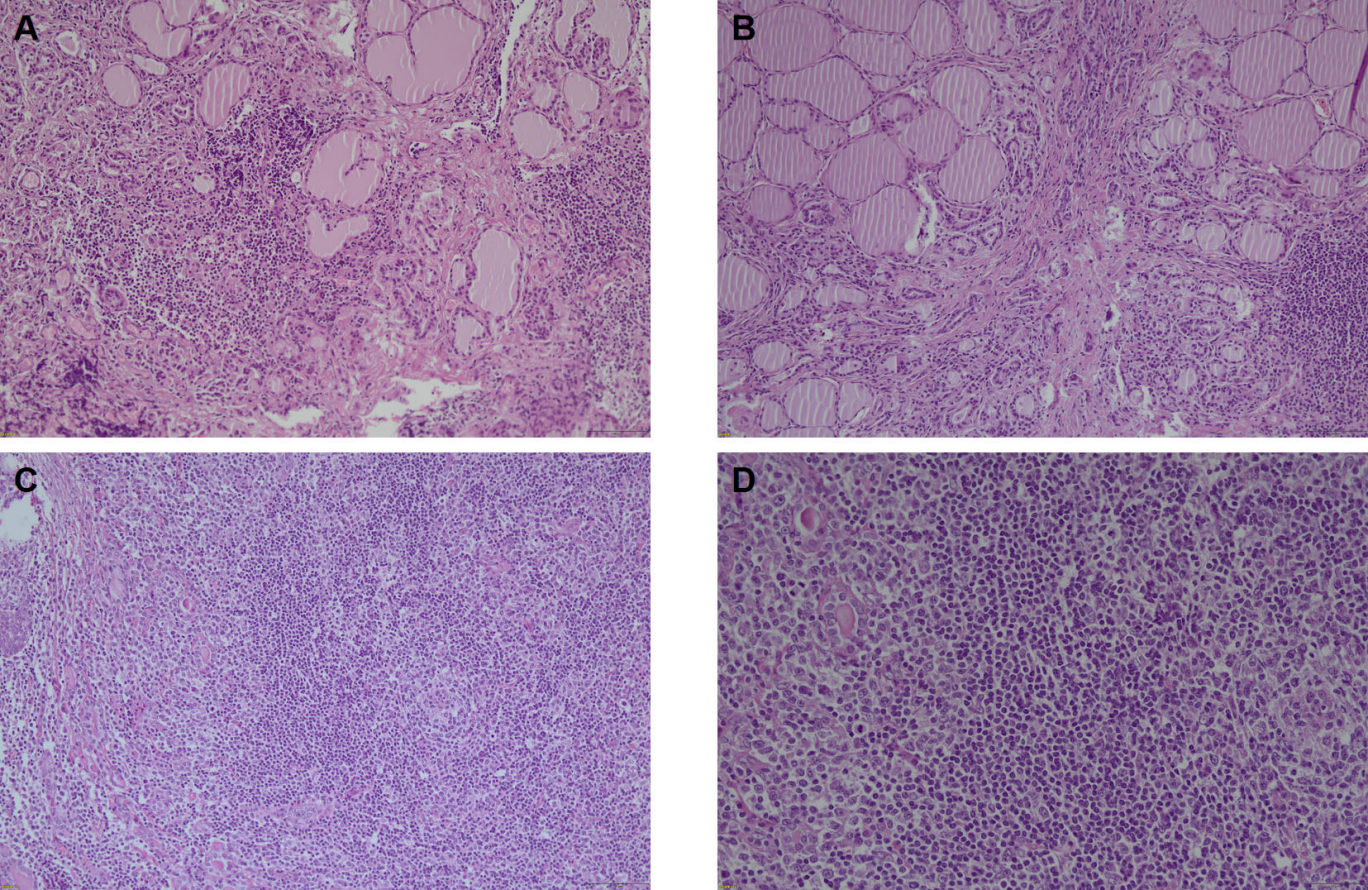
Histopathological findings of Hashimoto thyroiditis, papillary thyroid microcarcinoma, and primary thyroid lymphoma. **(A)** Hematoxylin-eosin staining showing Hashimoto thyroiditis (original magnification×20). **(B)** Hematoxylin-eosin staining showing papillary thyroid microcarcinoma (original magnification ×20). **(C)** Hematoxylin-eosin staining showing primary thyroid lymphoma (PTL)(original magnification ×20). **(D)** Hematoxylin-eosin staining showing PTL (original magnification×40).

**Figure 3:**
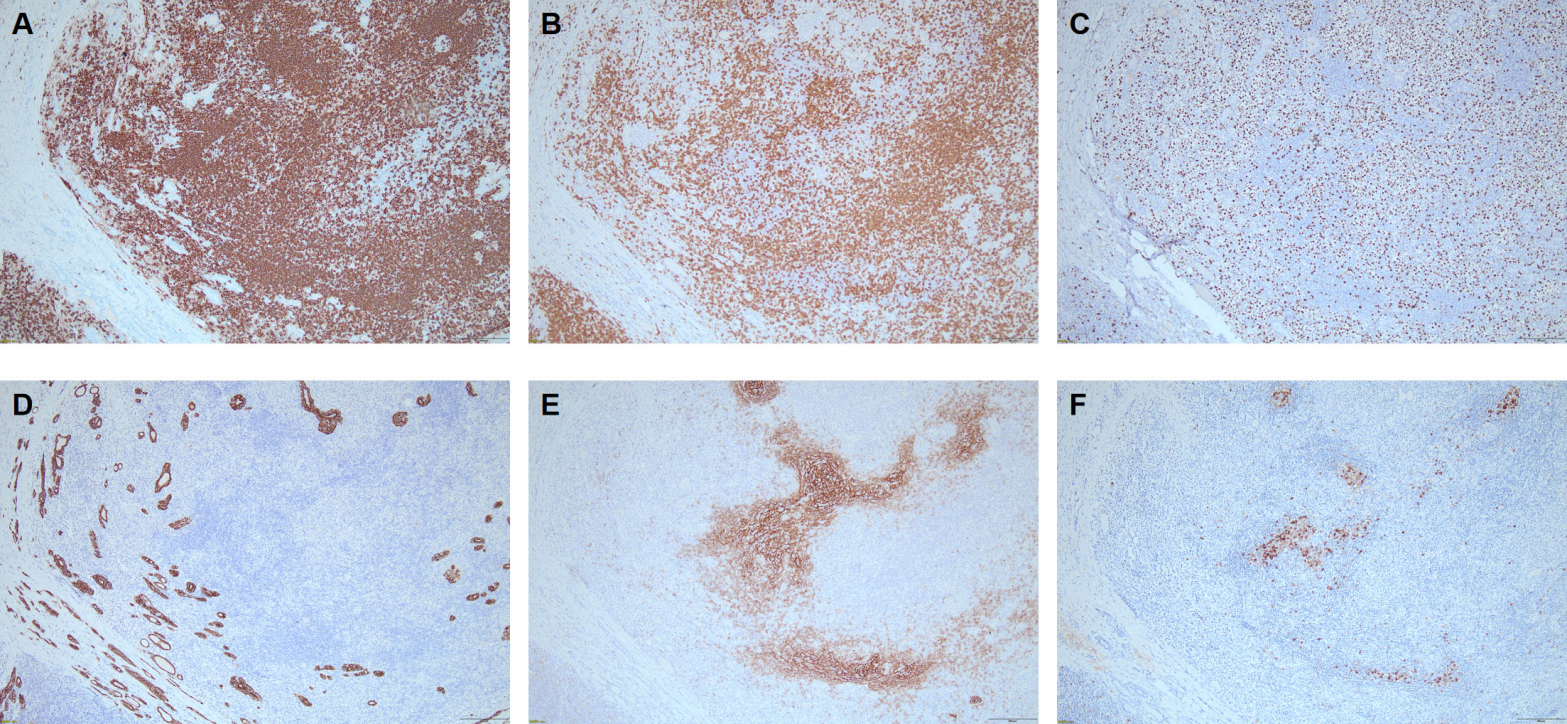
Immunohistochemical characterization of the thyroid lymphoma component. Immunohistochemistry assay showing the expression of **(A)** CD20, **(B)** CD3, **(C)** Ki-67, **(D)** CK, **(E)** CD21, and **(F)** CD10 in primary thyroid lymphoma (PTL) lesions. CD20 positivity supported B-cell lineage, CD3 mainly highlighted background/reactive T cells, Ki-67 showed an increased proliferation index, CK helped distinguish the lymphoid lesion from epithelial carcinoma, CD21 highlighted follicular dendritic cell networks, and CD10 negativity contributed to the immunophenotypic classification.

Therefore, right thyroid lobectomy with isthmusectomy and sampling of the suspicious right level VI lymph node was selected as a diagnostic and therapeutic procedure. Intraoperative frozen-section examination suggested lymphocytic thyroiditis with fibrosis, and immediate conversion to total thyroidectomy was not performed. On the first postoperative morning, parathyroid hormone and serum calcium levels were measured at 52.40 pg/mL (reference range, 15.0-68.3) and 2.21 mmol/L (reference range, 2.20-2.65), respectively. The patient experienced no complications, such as hoarseness or dysphagia. A definitive histopathological examination of the paraffin-embedded sections revealed, against a background of HT (**Figure 2A**), PTMC in the right lobe and the isthmus (maximum diameter, 2 mm) (**Figure 2B**). Carcinoma was identified in one of ten resected right level VI (central compartment) lymph nodes (1/10). Concurrently, a non-Hodgkin B-cell lymphoma was identified in the thyroid lesion. The postoperative pathological report supported extranodal marginal zone lymphoma of mucosa-associated lymphoid tissue type (MALT lymphoma) and described pathological features suggestive of large B-cell transformation (**Figures 2C, D**). Immunohistochemical analysis yielded positive CD20 expression in the follicular areas, indicating B-cell lineage lymphoma (**Figure 3A**). Negative CD3 staining ruled out a T-cell origin (**Figure 3B**). The Ki-67 proliferation index was 40% (**Figure 3C**). Negative cytokeratin staining indicated the absence of keratinization (**Figure 3D**). CD21 positivity highlighted abnormal follicular dendritic cell networks (**Figure 3E**). Negative CD10 expression in the lymphoma suggested a residual germinal center pattern (**Figure 3F**). Additional markers included CD79a(+), Pax-5(+), CD43 (–), CD30(+30%), CD138 (–), Bcl-6 (–), Bcl-2(+), CD5 (–), Cyclin D1 (–), SOX-11 (–), CD23(DC+), CD19(+), CD38(+), LMO2 (–), P53 (wild-type expression pattern), ALK (–), c-Myc(+10%), Mum-1(+), and MPO (–). *In situ* hybridization for Epstein-Barr virus-encoded RNA was negative in the tumor (positive control). The B-cell gene rearrangement was positive. After hematology consultation, the disease was clinically diagnosed and managed as primary thyroid MALT lymphoma, with the pathological large-cell transformation feature taken into consideration during treatment planning and follow-up. These findings collectively confirmed the coexistence of primary thyroid MALT lymphoma and PTMC with level VI lymph node metastatic carcinoma.

Postoperative systemic staging was performed under the guidance of the hematology team. PET-CT performed on April 15, 2024, approximately one month after surgery, showed postoperative changes in the operative bed and heterogeneous FDG uptake in the residual thyroid tissue, with several small cervical and left supraclavicular lymph nodes showing mild FDG uptake; follow-up was recommended. No definite distant hypermetabolic lesion was identified. Bone marrow evaluation performed on June 4, 2024, showed no evidence of bone marrow involvement by lymphoma.

Considering the clinical diagnosis of primary thyroid MALT lymphoma, pathological features suggestive of large-cell transformation, postoperative staging findings, absence of bone marrow involvement, and the patient’s preference, single-agent obinutuzumab was administered as hematology-directed anti-CD20 therapy. The patient received four cycles, comprising six infusions, and tolerated the treatment well. For the PTMC component, postoperative thyroid hormone therapy was administered during the first postoperative year with an intended TSH-suppression strategy, and thyroid function was monitored regularly. The last thyroid-related follow-up was performed on December 20, 2024, and thyroid function indices were within the acceptable range without clinically evident thyroid dysfunction. For the lymphoma component, the last hematology follow-up was performed on March 27, 2025. Thyroid and superficial lymph node ultrasonography showed no abnormal findings in the thyroid region, cervical lymph nodes, or axillary lymph nodes. Laboratory tests showed normal lactate dehydrogenase (LDH, 176 U/L), normal β2-microglobulin (1.75 mg/L), and a largely normal complete blood count, with red blood cell, white blood cell, and platelet counts within the reference ranges. At the last available follow-up, there was no clinical, ultrasonographic, or laboratory evidence of recurrence.

## 3 Discussion

HT likely provides a shared pathological background for both primary thyroid lymphoma and PTC. Chronic autoimmune thyroiditis is characterized by persistent lymphocytic infiltration and lymphoid follicle formation within the thyroid gland, creating a microenvironment that may favor the development of thyroid MALT lymphoma (8). Previous studies have also suggested that chronic antigenic stimulation, NF-κB-related molecular alterations, and autoimmune thyroiditis-associated immune dysregulation may contribute to MALT lymphomagenesis (19, 20). In parallel, HT has been reported to coexist with PTC/PTMC, although its association with tumor progression remains complex and may involve both detection bias and biological interactions (9, 10, 21, 22). In the present case, the coexistence of HT, thyroid MALT lymphoma, and PTMC complicated the preoperative interpretation of the thyroid lesion and highlighted the need for careful pathological assessment.

Currently, there is no reliable standalone method for diagnosing PTL. Its sonographic features often resemble those of HT, leading to frequent misdiagnoses or oversight. Rapidly enlarging sonographically atypical nodules in patients with HT should raise the suspicion of a coexisting malignancy. Although fine-needle aspiration (FNA) offers the advantages of simplicity and safety and is the gold standard for diagnosing differentiated thyroid carcinoma, its true accuracy for PTL remains debated. A retrospective series reported diagnostic sensitivities ranging from 39% to 56%. This limited sensitivity is primarily attributed to insufficient sample quantity obtained via FNA, as well as variability influenced by the operator’s experience and the cytopathologist’s interpretation of the specimen (23). Core needle biopsy can obtain a larger tissue sample and preserve intact tissue architecture, often resulting in higher sensitivity and diagnostic efficacy than FNA. In recent years, advancements in biopsy techniques and ancillary diagnostic methods have enabled the definitive preoperative diagnosis of many lymphomas. However, surgical excisional biopsy remains necessary for definitive diagnosis in certain complex scenarios. In the present case, lymphoma was not suspected preoperatively, and the patient declined to undergo preoperative FNA. Consequently, a definitive diagnosis was established through postoperative pathological examination using immunohistochemistry.

Currently, there is no established literature on the optimal management of concurrent PTL and PTC. Surgical resection is the standard treatment for PTC. In this case, the preoperative ultrasonographic findings were highly suggestive of primary thyroid carcinoma with lymph node metastasis, which is one of the common indications for thyroid surgery. The initial procedure addressed the highly suspicious right thyroid lesion and allowed pathological assessment of the suspicious central compartment lymph node. After the final pathological diagnosis, management required an individualized multidisciplinary approach that considered both the thyroid carcinoma component and the lymphoma component. The decision regarding whether to pursue completion surgery or adopt a non-surgical strategy required careful consideration of the postoperative pathological findings, systemic staging results, potential benefits and risks, and the patient’s preference. Postoperative PET-CT and bone marrow evaluation were performed to assess systemic involvement, and the absence of definite distant hypermetabolic disease or bone marrow involvement supported the subsequent individualized hematology-directed treatment strategy. After discussion with the patient and her family, local radiotherapy and anti-CD20 monoclonal antibody therapy were discussed as postoperative options. The patient chose anti-CD20 monoclonal antibody therapy and received four cycles, comprising six infusions, of single-agent obinutuzumab, together with a plan for long-term surveillance. Regular high-frequency neck ultrasonography was recommended for monitoring the residual left thyroid lobe and cervical lymph nodes. For the PTMC component, postoperative thyroid hormone therapy with an intended TSH-suppression strategy was adopted during the first postoperative year. Periodic systemic evaluation was planned for lymphoma surveillance.

Of particular note was the presence of level VI CLNM. Given the coexistence of PTMC and thyroid MALT lymphoma, the nodal lesion required careful pathological interpretation. PTMC can metastasize through lymphatic pathways, and the central compartment is a common site of nodal involvement (24). In contrast, MALT lymphoma may involve regional lymph nodes, but this is generally described as nodal involvement or local dissemination rather than carcinoma-type metastatic deposits, and involved lymph nodes often show lymphomatous architectural effacement (25, 26). In this case, postoperative pathology supported metastatic carcinoma from the PTMC component rather than lymphomatous nodal involvement.

PTMC generally has an excellent prognosis, and active surveillance is an accepted option for carefully selected low-risk patients (27). Nevertheless, nodal metastasis is a recognized event in a subset of PTMCs. In this case, the carcinoma component measured only 2 mm, whereas one right level VI lymph node contained metastatic carcinoma. This discrepancy between the very small primary tumor size and the presence of nodal metastasis highlights the need to evaluate risk according to the complete clinicopathological context rather than tumor size alone. Therefore, the central message of this case is not that PTMC should be regarded as biologically aggressive, but that suspicious central compartment lymph nodes should be carefully assessed and postoperative surveillance should be individualized.

According to postoperative pathology, the PTMC component was accompanied by metastasis in one right level VI lymph node and could be described as pT1aN1a. Because only one central compartment lymph node was involved, postoperative risk assessment should be individualized by considering the size of the nodal metastatic deposit, the presence or absence of extranodal extension, completeness of resection, serum thyroglobulin/anti-thyroglobulin antibody status when available, and follow-up imaging findings. In this context, the preoperatively detected suspicious level VI lymph node should be interpreted as an important clinical warning sign that supported careful nodal assessment and risk-adapted postoperative surveillance, rather than as direct evidence of macroscopic nodal disease.

Based on this individualized risk assessment, postoperative thyroid hormone therapy with an intended TSH-suppression strategy was adopted during the first postoperative year, together with regular ultrasonographic and thyroid function monitoring.

Ki-67 is a nuclear protein associated with cellular proliferation. Its index reflects the percentage of tumor cells in the active phase of the cell cycle (28). In indolent lymphomas, such as typical MALT lymphomas, the Ki-67 index is generally low, typically ranging between 5% and 20%. In contrast, aggressive lymphomas, such as DLBCL, usually exhibit a high Ki-67 index, frequently exceeding 40-80%. In the present case, the Ki-67 index of the MALT lymphoma component was as high as 40%, which was significantly higher than the expected range for typical indolent lymphoma. This finding indicates the high proliferative activity of the tumor cells and suggests a potential trend toward transformation to a higher-grade lymphoma. This interpretation aligns with the postoperative pathological description of features suggestive of large-cell transformation. Rearrangement of c-MYC and complete or partial inactivation of p53 may influence the transformation of MALT lymphoma to DLBCL (25). To interpret the elevated Ki-67 index, we carefully considered the possibility of contribution from the PTMC component. However, based on morphological localization and immunohistochemical analysis, we concluded that it more likely reflects the highly proliferative state of MALT lymphoma itself. This assessment provides a crucial rationale for selecting individualized treatment regimens.

Both PTMC and thyroid MALT lymphoma are often associated with relatively favorable clinical courses when diagnosed at an early stage. However, management should be based on the complete clinicopathological profile rather than diagnostic labels alone. In the present case, this included a very small PTMC with central lymph node metastasis, primary thyroid MALT lymphoma with pathological features suggestive of large-cell transformation, postoperative systemic staging, bone marrow assessment, and the patient’s treatment preference. These factors supported individualized multidisciplinary management and careful long-term surveillance.

This case has several limitations. First, preoperative FNA or core needle biopsy was not performed because the patient declined biopsy, which limited preoperative diagnostic certainty and may have influenced the initial surgical strategy. Second, PET-CT was performed in the early postoperative period, and local FDG uptake in the operative bed and residual thyroid tissue should therefore be interpreted cautiously. Third, although bone marrow evaluation did not show lymphoma involvement and the latest follow-up showed no clinical, ultrasonographic, or laboratory evidence of recurrence, longer follow-up is still required to determine the durability of disease control. Finally, as a single case report, the findings cannot be generalized, but they highlight the diagnostic complexity of suspicious thyroid lesions in the setting of Hashimoto thyroiditis.

## 4 Conclusion

This case report presents the exceedingly rare coexistence of HT, primary thyroid MALT lymphoma with pathological features suggestive of large-cell transformation, and lymph node metastatic PTMC. This case emphasizes the importance of preoperative tissue diagnosis when feasible, careful evaluation of suspicious cervical lymph nodes, precise histopathological and immunophenotypic classification, postoperative staging, and individualized multidisciplinary management. Although PTMC generally has an excellent prognosis, nodal metastasis may occur in a subset of patients, supporting risk-adapted postoperative surveillance.

## 5 Patient perspective

After surgery, learning that I had both thyroid carcinoma and lymphoma caused considerable anxiety. The explanations provided by the thyroid surgery, pathology, and hematology teams helped me understand why further treatment and long-term follow-up were needed. After completing postoperative therapy and receiving reassuring follow-up evaluations, I felt more confident about my condition and appreciated the multidisciplinary care I received.

## Data Availability

The original contributions presented in the study are included in the article/**Supplementary Material**. Further inquiries can be directed to the corresponding authors.
