# Supplementary figures and images for: Case report: Concurrent primary thyroid MALT lymphoma and lymph node metastatic thyroid micropapillary carcinoma in Hashimoto’s thyroiditis: a diagnostic and therapeutic challenge
Source: Front Oncol. 2026 Jul 13;16:1848147. doi: 10.3389/fonc.2026.1848147 (PMC13402122; doi:10.3389/fonc.2026.1848147)

# Time Line

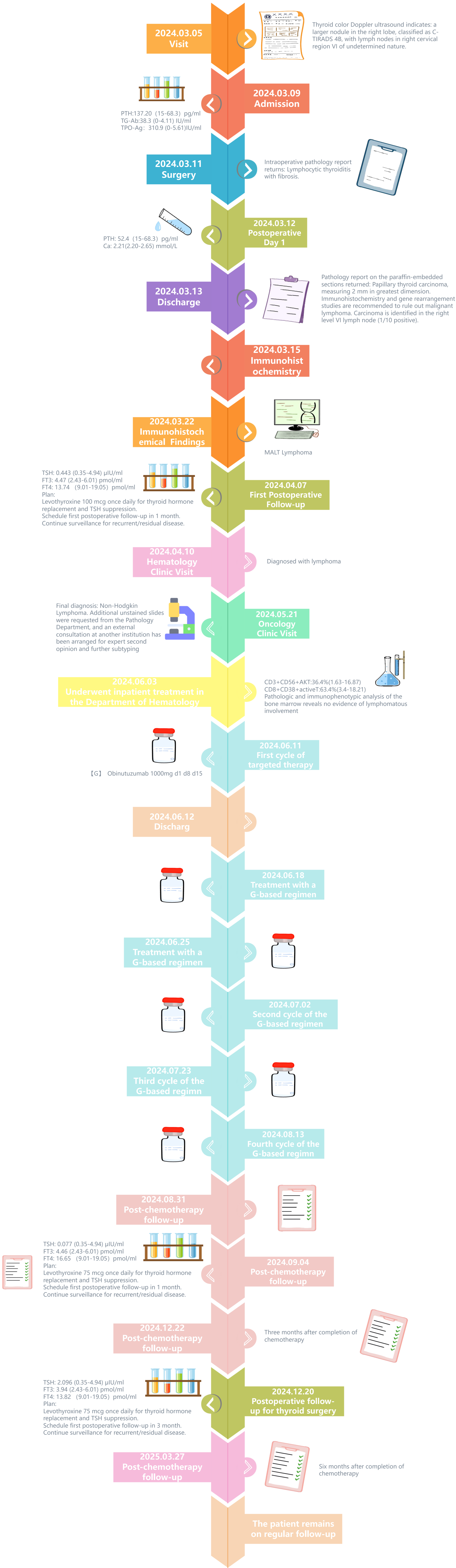

Supplement: Supplementary file 1 [file DataSheet1.pdf]
