# Supplementary material for: Case report: Concurrent primary thyroid MALT lymphoma and lymph node metastatic thyroid micropapillary carcinoma in Hashimoto’s thyroiditis: a diagnostic and therapeutic challenge
Source: Front Oncol. 2026 Jul 13;16:1848147. doi: 10.3389/fonc.2026.1848147 (PMC13402122; doi:10.3389/fonc.2026.1848147)
Supplement: Supplementary file 2 [file Table1.docx]

**Table S1. Published case reports/case series of coexisting primary thyroid MALT lymphoma and thyroid carcinoma retrieved mainly.**

| **First author/year** | **Age/sex** | **Thyroid function or HT/thyroiditis** | **Lymphoma subtype** | **PTC/PTMC** | **LN involvement** | **Imaging/pathology information** | **Treatment** | **Follow-up/outcome** |
| --- | --- | --- | --- | --- | --- | --- | --- | --- |
| de Melo GM, et al / 2010.(21) | NA | NA | Thyroid MALT lymphoma | PTC | NA | Only confirmed in the present review as a coexisting thyroid MALT lymphoma and PTC case report. | NA | NA |
| Cheng V, et al / 2012.(22) | NA | HT | Primary thyroid lymphoma | PTC | NA | NA | NA | NA |
| Nam YJ, et al / 2013.(23) | 81 / F | HT | MALT | PTC | NA | Initial FNA suggested PTC; coexisting MALT lymphoma was recognized subsequently. | Diagnosis established after surgery; surgical procedure not reported. | NA |
| Tarui T, et al / 2014.(24) | NA | NA | MALT | PTC | NA | NA | Emergency total thyroidectomy. | NA |
| Shen G, et al / 2015.(25) | 25 / F | HT present; FNA background of lymphocytic thyroiditis. | MALT | PTC | NA | FNA showed atypical follicular epithelial cells and lymphoid cells. | Total thyroidectomy + chemotherapy + RAI. | No recurrence after 2 years; Tg was undetectable. |
| Lan XB, et al / 2018.(26) | NA | HT | MALT | PTC | NA | NA | NA | NA |
| Zhang J, et al / 2024.(27) | NA | NA | Primary thyroid MALT lymphoma | PTC | NA | The abstract highlights that lymphoma was not suspected preoperatively; PMC full text was identified. | NA | NA |
| Babaya N, et al / 2025.(28) | 58 / M | History of autoimmune/Hashimoto thyroiditis. | MALT | PTC | NA | The abstract reports 6 months of hoarseness and thyroid enlargement. | NA | NA |

Abbreviations: F, female; FNA, fine-needle aspiration; HT, Hashimoto thyroiditis; LN, lymph node; M, male; MALT, mucosa-associated lymphoid tissue; NA, not available/not reported in the accessible source; PTC, papillary thyroid carcinoma; PTMC, papillary thyroid microcarcinoma; RAI, radioactive iodine; Tg, thyroglobulin. Age is reported in years.

Note: This table is intended as an evidence-map/literature-synthesis table for manuscript submission.
